# The impact of COVID‐19 on clinical outcomes among acute myocardial infarction patients undergoing early invasive treatment strategy

**DOI:** 10.1002/clc.23908

**Published:** 2022-08-30

**Authors:** Prerna Sharma, Kajal Shah, Johanna Loomba, Arti Patel, Indika Mallawaarachchi, Olivia Blazek, Sarah Ratcliffe, Khadijah Breathett, Amber E. Johnson, Angela M. Taylor, Michael Salerno, Michael Ragosta, Nishtha Sodhi, Daniel Addison, Selma Mohammed, Kenneth C. Bilchick, Sula Mazimba

**Affiliations:** ^1^ Division of Cardiovascular Medicine University of Virginia Medical Center Charlottesville Virginia USA; ^2^ Department of Internal Medicine University of Virginia Medical Center Charlottesville Virginia USA; ^3^ Integrated Translational Health Research Institute (iTHRIV) University of Virginia Charlottesville Virginia USA; ^4^ Department of Public Health Sciences University of Virginia Charlottesville Virginia USA; ^5^ Division of Cardiology University of Connecticut—Hartford Hospital Mansfield Connecticut USA; ^6^ Division of Cardiology University of Arizona Medical Center Tucson Arizona USA; ^7^ Division of Cardiology, University of Pittsburgh Medical Center Pittsburgh Pennsylvania USA; ^8^ Division of Cardiology, Ohio State University Wexner Medical Center Columbus Ohio USA; ^9^ Division of Cardiology Creighton University School of Medicine Omaha Nebraska USA

**Keywords:** cardiac catheterization, COVID‐19, pediatric clinical cardiology, percutaneous coronary intervention

## Abstract

**Background:**

The implications of coronavirus disease 2019 (COVID‐19) infection on outcomes after invasive therapeutic strategies among patients presenting with acute myocardial infarction (AMI) are not well studied.

**Hypothesis:**

To assess the outcomes of COVID‐19 patients presenting with AMI undergoing an early invasive treatment strategy.

**Methods:**

This study was a cross‐sectional, retrospective analysis of the National COVID Cohort Collaborative database including all patients presenting with a recorded diagnosis of AMI (ST‐elevation myocardial infarction (MI) and non‐ST elevation MI). COVID‐19 positive patients with AMI were stratified into one of four groups: (1a) patients who had a coronary angiogram with percutaneous coronary intervention (PCI) within 3 days of their AMI; (1b) PCI within 3 days of AMI with coronary artery bypass graft (CABG) within 30 days; (2a) coronary angiogram without PCI and without CABG within 30 days; and (2b) coronary angiogram with CABG within 30 days. The main outcomes were respiratory failure, cardiogenic shock, prolonged length of stay, rehospitalization, and death.

**Results:**

There were 10 506 COVID‐19 positive patients with a diagnosis of AMI. COVID‐19 positive patients with PCI had 8.2 times higher odds of respiratory failure than COVID‐19 negative patients (*p* = .001). The odds of prolonged length of stay were 1.7 times higher in COVID‐19 patients who underwent PCI (*p* = .024) and 1.9 times higher in patients who underwent coronary angiogram followed by CABG (*p* = .001).

**Conclusion:**

These data demonstrate that COVID‐19 positive patients with AMI undergoing early invasive coronary angiography had worse outcomes than COVID‐19 negative patients.

## 1 INTRODUCTION

There is a growing body of evidence regarding the cardiovascular manifestations of coronavirus disease 2019 (COVID‐19) as well as the increased risk of cardiovascular morbidity and mortality.^1^, ^2^ These consequences may be related to the direct effects of the virus on the heart in addition to the pandemic's on healthcare systems leading to disruption in acute care pathways or alterations in health‐seeking behaviors.^3^ Hospitalized patients with COVID‐19 have a 10%–30% risk of myocardial injury, with higher rates in severe cases.^4^, ^5^, ^6^ This may be akin to the effects of other cardiotropic viruses that may induce direct myocardial injury or myocardial infarction.^7^ Multiple mechanisms have been proposed including activation of inflammatory cells within atherosclerotic plaques, generation of a prothrombotic state leading to coronary thrombosis, and increase in metabolic demands from an activated immune system.^6^

An early invasive strategy (24–72 h) for acute myocardial infarction (AMI) is associated with improved outcomes among high‐risk patients.^8^ Accordingly, consensus guidelines recommend an early invasive coronary angiogram with a revascularization strategy for patients presenting with high‐risk AMI.^9^ The most common pathways following an early invasive diagnostic coronary angiogram include: (a) percutaneous coronary intervention (PCI) of the culprit vessel, (b) surgical revascularization, (c) a combination of surgical technique and PCI, and (d) medical management. The implications of COVID‐19 infection status on the outcomes after invasive therapeutic strategies among patients presenting with AMI is not well studied. We, therefore, evaluated the association of COVID‐19 positive status with adverse outcomes among patients presenting with AMI (ST‐elevation AMI and non‐ST elevation AMI) undergoing coronary angiography within 72 h of presentation to the hospital. We tested the hypothesis that there would be differential outcomes in COVID‐19 patients who underwent coronary angiograms for AMI as compared to COVID‐19 negative patients.

## 2 METHODS

### 2.1 Study design and participants

This study was a cross‐sectional, retrospective analysis of the National COVID Cohort Collaborative (N3C) database and was approved by the Institutional Review Board (IRB) at the University of Virginia Hospital Center. Due to the retrospective study design, the IRB waived the need for written patient informed consent. The N3C^10^ database includes electronic health record data from healthcare institutions across the United States. The N3C data transfer to NCATS is performed under a Johns Hopkins University Reliance Protocol #IRB00249128 or individual site agreements with NIH. The N3C Data Enclave is managed under the authority of the NIH; information can be found at https://ncats.nih.gov/n3c/resources. At the time of this analysis, 50 institutions had contributed data to N3C (spans from January 1, 2018, through April 10, 2021). Demographics were administratively reported. AMI events in the N3C data set spanned 2 years before the onset of the COVID‐19 pandemic. Severe acute respiratory syndrome coronavirus 2 (SARS‐COV‐2) diagnostics were still developing in the early part of 2020 and the diagnosis code (U07.1) was not introduced by the Centers for Disease Control until March 2020. Since coded data representing a positive test or diagnosis was not present in our data set until March 15, 2020, we used this date as the start date for our COVID‐19 era.

All patients in the study cohort with a SARS‐COV‐2 positive polymerase chain reaction (PCR) or antigen lab test or COVID‐19 diagnosis were flagged as COVID‐19 positive (COVID+) as of the date of the first positive test or first diagnosis (whichever was earliest). All other patients were considered COVID‐19 negative (COVID−) although we recognize it is possible that not all infections would have been detected and/or recorded in the medical record. Patients with COVID‐19 diagnosis within 3 months before or up to 2 days after the patient's first reported AMI (index AMI event) were included in the COVID‐19 positive AMI group. This window was used because of the established lingering effects of COVID‐19 symptoms and increased adverse cardiovascular effects following the index infection.^11^, ^12^ All patients with ST‐elevation AMI (STEMI) and non‐ST elevation AMI (NSTEMI) were included.

We then stratified the COVID‐19 POSITIVE AMI patients into four groups that represent the current therapeutic paradigms arising from a diagnostic coronary angiogram. We included patients that underwent an early invasive treatment strategy within 72 h of diagnosis and who had a corresponding reported treatment in the N3C database. Patients that did not have complete documented therapies in the N3C database for their AMI were excluded. The four groups are as follows:

1) Treatment Group 1: Coronary angiogram with a PCI within 3 days of AMI
  a) without coronary artery bypass graft (CABG) within 30 days following AMI (PCI)
  b) with CABG within 30 days following AMI (PCI with CABG)
2) Treatment Group 2: Coronary angiogram without PCI within 3 days of AMI
  a) without CABG within 30 days following AMI (neither PCI nor CABG)
  b) with CABG within 30 days following AMI (CABG).

Given that the treatment guidelines for acute coronary syndromes recommend consideration for early risk stratification of AMI with coronary angiogram and or revascularization within 72 h, our cohort was constructed to capture patients who were considered for this treatment strategy. To evaluate whether our analysis should include control patients from the pre‐COVID‐19 era, we evaluated treatment groups' pre and post‐COVID‐19 era. We detected a variation in treatment groups between these two time periods (*p* < .001). As a result, we only included COVID‐19− patients (comparison group) who had their initial AMI event during the COVID‐19 era.

Groups of relevant medical codes were identified using the Observation Medical Outcomes Partnership (OMOP) common data model. These groups of medical codes, or concept sets, were created to identify COVID‐19 diagnoses, AMI, as well as all criteria (comorbidities and outcome variables) used for matching. These concept sets were created using an open‐source application called Atlas. Since sites use different medical coding, we only used codes standardized by the OMOP common data model to define our patient cohort and our variables because the N3C database also uses the OMOP common data model. Since Atlas allows us to collect standardized codes, we used this tool toto match the same data model as N3C. While many concept sets were custom built for this analysis, there were four concept sets that were already created that were reused to construct the data set for this analysis. Concept sets to identify cancer diagnoses and chronic obstructive pulmonary disease (COPD) were used in the N3C Consortium publication^13^ on COVID‐19 early severity prediction. The full list of concept sets is provided in supplemental materials, along with links to the code workbooks for reproducibility of variable derivation and statistical analysis (eMethods in the Supporting Information).

### 2.2 Propensity matching

Data set was stratified into four groups based on the treatment received. For each strata, the propensity score for COVID‐19 positivity was calculated via logistic regression with independent variables including demographics (age, sex, race, ethnicity), data partner ID, smoking status, and diagnosis of certain comorbidities on or before their first reported AMI. These comorbidities included diabetes type II, hyperlipidemia, uncontrolled hypertension, controlled hypertension, heart failure with reduced ejection fraction, heart failure preserved ejection fraction, peripheral arterial disease, obesity, atrial fibrillation/flutter, COPD, coronary artery disease (CAD), CABG procedures, cancer, and history of stroke. Height and weight measurements were also used to calculate body mass index (BMI) so that any patients with a BMI over 30 kg/m^2^ on the measurement date most recent to their AMI were considered obese. Exact matching was done based on race and data partner ID, while nearest neighbor matching was done based on other variables. Demographic characteristics of patients pre‐ and postmatching according to treatment group are outlined in  Supporting Information: Table S1. COVID‐19 positive patients were matched to COVID‐19 negative in a 1:3 ratio, using a calliper of 0.2 (Table 1). Only AMI (STEMI and NSTEMI) patients after March 15, 2020 were included in the study to ensure comparisons between the two groups (COVID‐19 positive vs. negative) were made during the prevailing conditions of the pandemic.

**Table 1:** Descriptive characteristics of COVID‐positive (+) and COVID‐negative (−) patients as per invasive treatment strategy

	PCI		PCI with CABG		Neither PCI nor CABG		CABG	
	COVID+, *N* = 173 (%)	COVID−, *N* = 397 (%)	COVID+, *N* = 69 (%)	COVID−, *N* = 133 (%)	COVID+, *N* = 222 (%)	COVID−, *N* = 540 (%)	COVID+, *N* = 60 (%)	COVID−, *N* = 130 (%)
Demographics								
Male	118 (68.0)	274 (69.0)	56 (81.0)	106 (80.0)	133 (60.0)	297 (55.0)	53 (88.0)	110 (85.0)
Female	55 (32.0)	123 (31.0)	<20	<20	89 (40.0)	243 (45.0)	<20	<20
White, non‐Hispanic	117 (68.0)	313 (79.0)	45 (65.0)	110 (83.0)	136 (61.0)	369 (68.0)	46 (77.0)	112 (86.0)
Black or African American	23 (13.0)	38 (10.0)	<20	<20	48 (22.0)	109 (20.0)	<20	<20
Hispanic or Latino	23 (13.0)	38 (10.0)	<20	<20	27 (12.0)	42 (8.0)	<20	<20
Asian, non‐Hispanic	<20	<20	0	0	<20	<20	<20	<20
Other, non‐Hispanic	0	0	0	0	<20	<20	0	0
Comorbidities								
Atrial fibrillation	30 (17.3)	74 (18.6)	<20	<20	54 (24.3)	135 (25.0)	<20	<20
Coronary artery disease	143 (82.7)	333 (83.9)	61 (88.4)	117 (88.0)	131 (59.0)	309 (57.2)	55 (91.7)	120 (92.3)
Cancer	<20	<20	<20	<20	22 (9.9)	44 (8.1)	<20	<20
COPD	61 (35.3)	131 (33.0)	22 (31.9)	35 (26.3)	75 (33.8)	176 (32.6)	<20	<20
Current smoker	37 (21.4)	100 (25.2)	<20	<20	40 (18.0)	96 (17.8)	<20	<20
Former smoker	<20	<20	<20	<20	<20	<20	<20	<20
Diabetes	86 (49.1)	178 (44.8)	37 (53.6)	58 (43.6)	95 (42.7)	216 (40.0)	32 (53.3)	58 (44.6)
HFpEF	26 (15.0)	67 (16.9)	<20	<20	45 (20.3)	108 (20.0)	<20	<20
HFrEF	35 (20.2)	85 (21.4)	<20	<20	73 (32.9)	175 (32.4)	<20	<20
History of CABG	<20	<20	49 (21.0)	98 (73.7)	<20	<20	<20	<20
Hyperlipidemia	134 (77.5)	312 (78.6)	47 (68.1)	95 (71.4)	131 (59.0)	314 (58.1)	52 (86.7)	107 (82.3)
Hypertension, controlled	129 (74.6)	297 (74.8)	45 (65.2)	90 (67.7)	145 (65.3)	351 (65.0)	46 (76.7)	97 (74.6)
Hypertension, uncontrolled	<20	<20	<20	<20	<20	<20	<20	<20
Obesity	90 (52.0)	221 (55.7)	27 (39.1)	47 (35.3)	120 (54.1)	283 (52.4)	32 (53.3)	71 (54.6)
Pulmonary artery disease	0	0	0	0	0	0	<20	<20
Previous stroke	<20	<20	<20	<20	<20	<20	<20	<20
Outcomes								
Cardiogenic shock	<20	<20	<20	<20	<20	<20	<20	<20
Respiratory failure	<20	<20	<20	<20	<20	<20	<20	<20
Prolonged LOS	69 (39.9)	97 (24.4)	<20	<20	76 (34.2)	115 (21.3)	36 (60.0)	76 (58.5)
Rehospitalization	31 (17.9)	79 (19.9)	<20	<20	54 (24.3)	99 (18.3)	<20	<20
Death	<20	<20	<20	<20	27 (12.2)	42 (7.8)	<20	<20

Abbreviations: CABG, coronary artery bypass graft; COPD, chronic obstructive pulmonary disease; COVID, coronavirus disease; HFpEF, heart failure with preserved ejection failure; HFrEF, heart failure with reduced ejection fraction; LOS, length of stay; PCI, percutaneous coronary intervention.

### 2.3 Outcome variables

The outcome variables included in this study were reported death, respiratory failure within 30 days of their AMI, cardiogenic shock within 2 days of their AMI, rehospitalization within 30 days of their AMI, and prolonged length of stay. Death was defined as a reported death in the patient record. Respiratory failure was defined using a concept set consisting of intubation of the respiratory tract, controlled mandatory ventilation, and veno‐venous extracorporeal membrane oxygenation. If these procedures occurred within 30 days of the patient's index AMI event, then this was considered as having respiratory failure. Cardiogenic shock was defined as having a diagnosis of cardiogenic shock within 2 days of the index AMI event. Rehospitalization was defined as being readmitted within 30 days of the index AMI event. Prolonged length of stay was defined as length of stay more than 3, 7, 5, and 7 days for Groups 1a, 1b, 2a, and 2b respectively. Matched data set was used to identify the differences in outcomes between COVID‐19 positive and negative subjects. Even though the pandemic has disproportionately affected minority ethnicities,^14^ our study did not include outcomes based on ethnicity because the cohorts were assembled using propensity matching to investigate the impact of COVID‐19 infection on treatment strategies.

### 2.4 Statistical analysis

COVID‐19 positivity is a binary indicator of whether the patient had a SARS‐COV‐2 positive PCR or antigen lab test or COVID‐19 diagnosis within the 3 months before or up to 2 days after their index AMI event. Conditional logistic regression was used to model the binary outcomes. Outcome modeling was also done separately for each treatment group. Holm–Bonferroni adjustment multiple was performed across all models, and adjusted *p*‐values have been reported. Analyses were performed using R statistical software.

## 3 RESULTS

**Figure 1:**
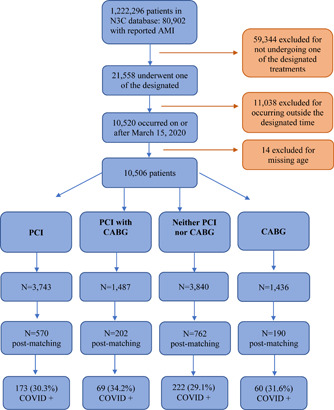
CONSORT flow diagram of the study. Out of the 10 506 patients included in the study, 570, 202, 762, and 190 patients were included after propensity patching in the PCI, PCI with CABG, neither PCI nor CABG, and CABG groups respectively. The number of COVID‐positive patients in all groups are depicted in the last row. AMI, acute myocardial infarction; CABG, coronary artery bypass graft; COVID, coronavirus disease; N3C, National COVID Cohort Collaborative; PCI, percutaneous coronary intervention.

The N3C registry included 1 222 296 adult patients of whom 10 520 had a diagnosis of AMI who met the prespecified inclusion criteria. After excluding 14 patients with no identified age, there were 10 506 patients included in the final cohort (Figure 1). The detailed descriptive results of demographics and comorbidities according to the invasive treatment strategy they received are displayed in Table 1.

**Figure 2:**
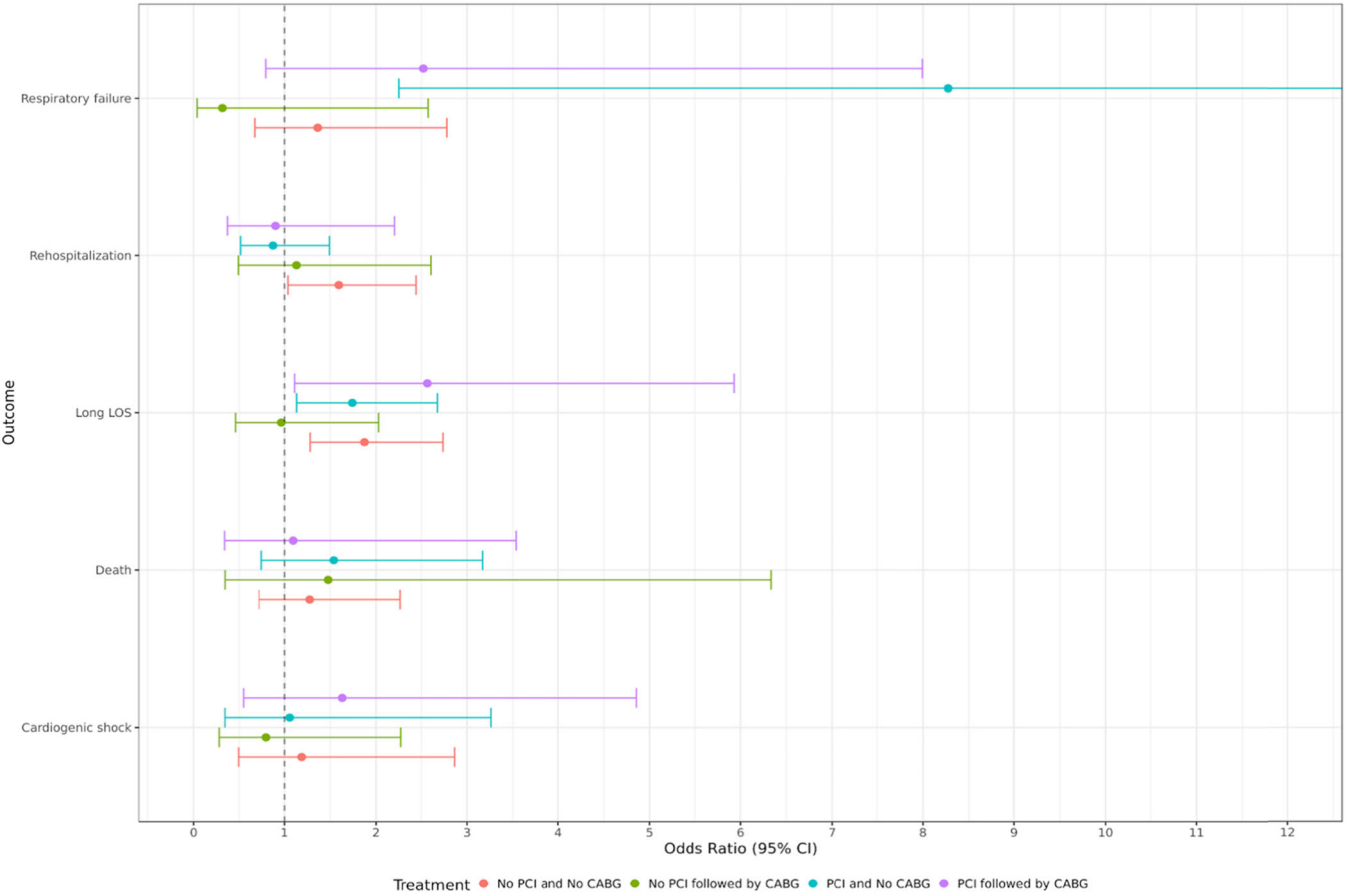
Odds of adverse outcomes in COVID‐19 positive versus negative subjects. The figure depicts the odds of respiratory failure, rehospitalization, long length of stay (LOS), death, and cardiogenic shock in COVID‐19 positive versus negative patients. COVID‐19‐positive patients who underwent PCI had higher odds of respiratory failure. The odds of prolonged length of stay were higher in COVID‐19 patients who underwent PCI and in patients who underwent CABG. CABG, coronary artery bypass graft; CI, confidence interval; COVID‐19, coronavirus disease 2019; PCI, percutaneous coronary intervention.

Of the 10 506 patients included in this study, 5% (524) with AMI tested positive for COVID‐19. Of the COVID‐19 positive patients, 16.4% (86) were non‐Hispanic African American, 11.6% (61) were Hispanic or Latino, and 67.4% (354) were non‐Hispanic White. There were no differences in the odds of death in any treatment group between COVID‐19 positive and negative patients. COVID‐19 positive patients who underwent PCI without CABG had 8.2 times higher odds of respiratory failure (odds ratio [OR] = 8.27; 95% confidence interval [CI] = 2.25–30.44; *p* = .001). The adjusted odds of prolonged length of stay was 1.7 times higher in COVID‐19 patients who underwent PCI without CABG (OR = 1.74; 95% CI = 1.13–2.68; *p* = .024) and 1.9 times higher in patients who underwent coronary angiogram followed by CABG (OR = 1.87; 95% CI = 1.28–2.74; *p* = .001). Detailed results of impact of COVID‐19 on treatment groups are displayed in Table 2 and Figure 2.

**Table 2:** Impact of COVID‐19 status in AMI on outcomes for different treatment groups in the matched sample

Outcomes in different treatment groups	*N* (%)		OR (95% CI)	Holm–Bonferroni adjusted *p*‐value
	COVID+	COVID−		
Mortality				
PCI	<20	<20	1.54 (0.75–3.17)	.97
PCI with CABG	<20	<20	1.09 (0.34–3.54)	.99
Neither PCI nor CABG	27 (12.16%)	42 (7.78%)	1.48 (0.34–6.33)	.99
CABG	<20	<20	1.27 (0.72–2.26)	.99
Respiratory failure				
PCI	<20	<20	8.27 (2.25–30.44)	.001
PCI with CABG	<20	<20	2.52 (0.80–7.99)	.60
Neither PCI nor CABG	<20	<20	0.31 (0.04–2.58)	.98
CABG	<20	<20	1.36 (0.67–2.78)	.99
Cardiogenic shock				
PCI	<20	<20	1.06 (0.34–3.26)	.99
PCI with CABG	<20	<20	1.63 (0.55–4.86)	.99
Neither PCI nor CABG	<20	<20	0.80 (0.28–2.27)	.99
CABG	<20	<20	1.19 (0.49–2.86)	.99
Rehospitalization				
PCI	31 (17.91%)	79 (19.90%)	0.87 (0.51–1.49)	.99
PCI with CABG	<20	<20	0.90 (0.37–2.20)	.99
Neither PCI nor CABG	54 (24.32%)	99 (18.33%)	1.13 (0.49–2.61)	.99
CABG	<20	<20	1.59 (1.04–2.44)	.13
Prolonged length of stay				
PCI	69 (39.88%)	97 (24.43%)	1.74 (1.13–2.68)	.024
PCI with CABG	<20	<20	2.57 (1.11–5.93)	.081
Neither PCI nor CABG	76 (34.23%)	115 (21.30%)	0.96 (0.46–2.03)	.99
CABG	36 (60.00%)	76 (58.46%)	1.87 (1.28–2.74)	.001

*Note*: OR and 95% CI are shown for COVID‐19 positive (+) versus COVID‐19 negative (−) patients.

Abbreviations: AMI, acute myocardial infarction; CABG, coronary artery bypass graft; CI, confidence interval; COVID‐19, coronavirus disease 2019; OR, odds ratio; PCI, percutaneous coronary intervention.

## 4 DISCUSSION

This study demonstrated that patients with COVID‐19 infection undergoing early invasive strategy for AMI had a higher rate of adverse events than those without COVID‐19 infection. More specifically, AMI patients with COVID‐19 who underwent PCI had a higher risk of respiratory failure as compared to COVID‐19− patients. Additionally, COVID‐19‐positive patients were more likely to have prolonged length of stay. It is crucial to note that even though patients with COVID‐19 and AMI had higher odds of death in all treatment groups, this was not statistically significant.

From this large cohort of AMI patients with COVID‐19 positive status, differences in outcomes appear to be driven at least in part by the adverse respiratory effects of the disease. Recent data from the North American COVID‐19 MI Registry have highlighted higher in‐hospital mortality and longer length of stay in COVID‐19 positive STEMI patients.^15^ There is limited data on the outcomes of AMI specifically based on the invasive treatment strategies. This study demonstrates the increased risk of respiratory failure in COVID‐19‐positive patients undergoing coronary angiograms with PCI for AMI (STEMI and NSTEMI). Additionally, our data reinforces the observed impact of the pandemic on the early decline in revascularization strategies of AMI.^3^, ^16^ There were variations noted in treatment strategies between pre‐ and post‐COVID‐19 era in MI patients. This may be attributed to practice changes that were implemented in the early phase of the pandemic to delay nonurgent/emergent procedures.

SARS‐CoV‐2 infection is not only associated with cardiac complications but patients with pre‐existing cardiovascular disease have been shown to have a greater risk of mortality.^1^, ^17^ There have been several proposed mechanisms causing cardiac involvement such as host receptor angiotensin‐converting enzyme 2 mediated cellular entry of virus, cytokine release and immune system activation leading to plaque instability.^18^, ^19^, ^20^ In addition, some reports have described a persistent residual risk of cardiovascular complications even after the initial infection has resolved that may continue to predispose patients to other cardiovascular events such as myocarditis.^12^, ^21^ Our study highlights the adverse effects of COVID‐19 on patients with CAD undergoing early invasive therapy with PCI. However, the subgroup of patients who underwent coronary angiograms without PCI or CABG did not have worse outcomes and they could potentially represent a heterogenous group of patients with myocarditis, stress cardiomyopathy, or CAD not amenable to interventions.

Overall, it is crucial to keep close vigilance on patients with AMI and concomitant respiratory viral infections. Studies have shown unfavorable outcomes in AMI patients with other respiratory viral infections such as influenza.^22^, ^23^ Cardoso et al.^22^ studied the therapies and outcomes of patients with concomitant AMI and influenza and demonstrated that patients with AMI and influenza had higher odds of in‐hospital mortality and multiorgan failure. Furthermore, the COVID‐19 pandemic has been associated with worse STEMI outcome metrics globally and COVID‐19 positive patients with STEMI represent a high‐risk group of patients.^15^, ^24^ Our study shows that patients with COVID‐19 infection and AMI (STEMI and NSTEMI) undergoing early invasive treatment have increased risk of adverse events such as respiratory failure and length of stay. In the North American COVID‐19 MI Registry, STEMI patients with COVID‐19 who did not undergo coronary angiography had higher mortality than those who did.^15^ Therefore, it is critically important to risk stratify patients with COVID‐19 and AMI to ensure appropriate invasive treatment strategies and optimize clinical outcomes.

### 4.1 Limitations

The limitations of this study include the real‐world effects of medical record data, N3C data selection pipeline, and informatics limitations pertaining to computational processes on this large data set. There could also potentially be unidentifiable discrepancies in the data due to inconsistency in codes entered by physicians across multiple institutions. Studies have demonstrated a decrease in hospitalizations for AMI during the pandemic.^16^ It would be reasonable to presume that the overall AMI admissions and interventions in our cohort were impacted by the pandemic. There is a wide range of false negative results based on the timing of the test. False negatives for PCR testing range from 20% to 100% while false negative for AG testing has been found to be <1%.^25^, ^26^ These real‐world limitations could not be mitigated in our analysis. Additionally, there were limitations related to the study design such as lack of subgroup analysis to assess outcomes in STEMI and NSTEMI patients separately. However, these limitations are offset by the robust, quality‐assured N3C data set which is the largest national database for COVID patients with centrally curate patient‐level data.^10^

## 5 CONCLUSION

Our data reveals the differences in outcomes and deleterious effects of COVID‐19 infection on AMI (STEMI and NSTEMI) patients undergoing early diagnostic coronary angiography. These data demonstrate that COVID‐19 positive patients with AMI undergoing early invasive coronary angiography had worse outcomes than COVID‐19 negative patients. The prolonged length of stay in COVID‐19‐positive patients has widespread implications on both patient outcomes and healthcare resource utilization. Timely diagnosis and intervention for AMI in this high‐risk group of patients is essential to assure appropriate allocation of treatment strategies to improve outcomes.

## CONFLICT OF INTEREST

The authors declare no conflict of interest.

## Supporting information

Supplementary information.

## Data Availability

The data that supports the findings of this study are available in the Supporting Information Material of this article and from the corresponding author upon reasonable request.
