# Supplementary material for: The impact of COVID‐19 on clinical outcomes among acute myocardial infarction patients undergoing early invasive treatment strategy
Source: Clin Cardiol. 2022 Aug 30;45(10):1070–8. doi: 10.1002/clc.23908 (PMC9538930; doi:10.1002/clc.23908)
Supplement: Supplementary file 1 — Supplementary information. [file CLC-45-1070-s001.docx]

**Supplementary Online Material**

eMethods: Supplementary Methods

eTable 1: Baseline Demographics Pre and Post Matching

**eMethods Supplemental Methods**

*Inclusion and Exclusion Criteria*

Acute myocardial infarction (AMI) patients who were treated using one of the following four treatment pathways were selected:

1. Cardiac catheterization **with a stent** within 3 days of their MI and **no** **CABG** within 30 days following MI
1a. Cardiac catheterization **with a stent** within 3 days of their MI, but **with CABG** within 30 days following MI
2. Cardiac catheterization **without a stent** within 3 days of their MI and **no CABG** in 30 days following MI
3. Cardiac catheterization **without a stent** within 3 days of their MI **with CABG** within 30 days following MI

AMI events in the N3C dataset spanned several years' time. In order to evaluate whether the analysis should include control patients from the pre-COVID era, the treatment arm assignments pre and post COVID era were evaluated. A variation in the treatment arm assignment between these two time periods was detected (p-value: <.001). Therefore, patients (COVID+ and control) who had their initial AMI event during the COVID-19 era were included. Patients who had their first reported AMI before March 15th, 2020 were excluded.

*Identifying Concept Codes and Code Workbook Used in the Creation of the Study Cohort*

The original code is available to N3C users who have been approved to access the private project DUR: [RP-E77CC5] Cardiovascular Complications of COVID-19.  The code workbook used for creating the person-level table of derived variable for the study cohort from raw N3C OMOP data is available here: <https://unite.nih.gov/workspace/vector/view/ri.vector.main.workbook.7b929cff-01b0-4bd2-bdbd-20157f07d9e0?branch=master>.  The code workbook used for the analysis of this table is available here: <https://unite.nih.gov/workspace/vector/view/ri.vector.main.workbook.a0c45942-bcb3-455d-9c04-a3dfb9ff2e90?branch=master>.  The report that reflects the data approved for extract for this publication is available here: <https://unite.nih.gov/workspace/report/ri.report.main.report.f3426b47-6f32-4c6f-9b10-e37908df6132>.

The variables used in the analyses represented data derived from N3C OMOP tables.  The data filtering was performed by first working with the clinicians and informaticists on our team to create OMOP “concept sets” (or “code sets”).  These concept sets represent groups of codes from any variety of medical coding vocabularies that the clinicians deemed appropriate for inclusion.  Because all data in N3C is mapped from original source codes to STANDARD OMOP codes, the enclave data was filtered using only standard codes.  The below list shows the standard codes used for our filters.  OHDSI tools such as ATLAS can be used to expand the below to identify all NON-STANDARD codes that map to the STANDARD OMOP codes listed.

*Concept Codes Used for Filters*

Acute Myocardial Infarction: 4119457, 4119943, 4121464, 4121465, 4124684, 4119948, 4126801, 46270162, 761737, 46270163, 45766076, 761736, 46270159, 46270160, 45766116, 45766151, 35611570, 35611571, 46274044, 46273495, 46270158, 46270164, 45766075, 4178129, 4267568, 4215140, 4011131, 44782769, 44782712, 45766115, 434376, 45766150, 438438, 4243372, 4108669, 4151046, 4275436, 438170, 45771322, 438447, 441579, 436706, 4324413, 4051874, 4303359, 4147223, 4189939, 4145721, 4119944, 4119456, 4119945, 4119946, 4121466, 4124685, 4270024, 35610091, 319039, 444406, 4119947, 3189643, 3188631, 4215259, 4173632, 4323202, 37309626, 4154704, 4200113, 4206867, 4124686, 765132, 45766113, 45773170, 4108677, 4108218, 45766241, 4172865, 439693

Cardiac Cath with Stent: 3181809, 3190422, 45760475, 4184832, 45768093, 36714929, 35614202, 35614209, 35614215, 35614198, 35614203, 35614210, 35614216, 35614221, 35614225, 35614204, 35614211, 35614217, 35614222, 35614199, 35614205, 35614212, 35614218, 35614223, 35614226, 35614206, 35614200, 35614207, 35614213, 35614219, 35614224, 35614227, 35614201, 35614208, 35614214, 35614220, 4031850, 45761737, 2725969, 2725470, 2725478, 42894627, 42894648, 42894660, 42894633, 42894654, 42894666, 2725464, 2725472, 2725480, 2725466, 2725474, 2725482, 42894625, 42894646, 42894658, 42894631, 42894652, 42894664, 42894623, 42894644, 42894656, 42894629, 42894650, 42894662, 725467, 2725475, 2725483, 2725968, 2725469, 2725477, 42894626, 42894647, 42894659, 42894632, 42894653, 42894665, 2725970, 2725471, 2725479, 2725465, 2725473, 2725481, 42894624, 42894645, 42894657, 42894630, 42894651, 42894663, 42894622, 42894643, 42894655, 42894628, 42894649, 42894661, 2725468, 2725476, 2725484, 2725897, 2725905, 2725913, 42894472, 42894484, 42894678, 42894478, 42894672, 42894684, 2725899, 2725907, 2725915, 2725901, 2725909, 2725917¸ 42894470, 42894482, 42894676, 42894476, 42894670, 42894682, 42894468, 42894480, 42894674¸ 42894474, 42894668, 42894680, 2725902, 2725910, 2725918, 2725896, 2725904, 2725912, 42894471, 42894483, 42894677, 42894477, 42894671, 42894683, 2725898, 2725906, 2725914, 2725900, 2725908, 2725916, 42894469, 42894481, 42894675, 42894475, 42894669, 42894681, 42894467, 42894479, 42894673, 42894473, 42894667, 42894679, 2725903, 2725911, 2725919, 2725945, 2725953, 2725961, 42894591, 42894603, 42894615, 42894597, 42894609, 42894621, 2725947, 2725955, 2725963, 2725949, 2725957, 2725965, 42894589, 42894601, 42894613, 42894595, 42894607, 42894619, 42894587, 42894599, 42894611, 42894593, 42894605, 42894617¸ 2725950, 2725958, 2725966, 2725944, 2725952, 2725960, 42894590, 42894602, 42894614, 42894596¸, 42894608, 42894620, 2725946, 2725954, 2725962, 2725948, 2725956¸2725964¸ 42894588, 42894600, 42894612, 42894594, 42894606, 42894618¸ 42894586, 42894598, 42894610, 42894592, 42894604, 42894616, 2725951, 2725959, 2725967, 2725921, 2725929, 2725937, 42894690¸ 42894702, 42894714, 42894696, 42894708, 42894585, 2725923, 2725931, 2725939, 2725925, 2725933, 2725941, 42894688, 42894700, 42894712, 42894694, 42894706, 42894718, 42894686, 42894698, 42894710, 42894692, 42894704, 42894716, 2725926, 2725934, 2725942, 2725920, 2725928¸ 2725936, 42894689, 42894701, 42894713¸ 42894695, 42894707, 42894584, 2725922¸ 2725930, 2725938, 2725924, 2725932, 2725940, 42894687, 42894699, 42894711, 42894693¸ 42894705, 42894717, 42894685, 42894697, 42894709, 42894691, 42894703, 42894715, 2725927, 2725935¸ 2725943¸ 417107, 4303797, 4330920¸ 45763528¸ 4328103, 4216356, 4238755, 3180695, 44789455, 2001504, 44511272, 44511133, 3188421, 43531439, 43531438, 43533247, 43533352, 45889355, 43527999, 43527998, 35607959, 44511268, 44511270, 44511269, 44511271, 45770795, 4020653, 4181025, 4006788, 4264285, 4265293, 4225903, 43527995, 43527994, 2313811, 2313810, 43527997, 43527996, 43533353, 43533248, 43527909, 43527908¸  2313804, 2313803, 45769224, 43531440, 45758408, 4178148, 4329263, 4283892, 37111313, 3173558, 3186322, 2107213¸ 3170433, 35614228, 35614229, 35614230, 35614231¸ 35614232, 35614233, 35614234, 35614235, 35614236, 35614237, 35614238, 35614239, 35614240-35614244, 35607668, 35614245-35614256, 45759254, 2617370, 2617369, 2313802, 2313801, 40756929

Cardiac Cath without Stent: 4219097, 35622857, 37394548, 37394549, 4085921, 4223020, 45765662, 4151830, 4236508, 4173410, 4284246, 4185904, 4218124, 4243461, 2001541, 40756852, 40757138, 40756911, 40756950, 40756979, 40756857, 40756944, 40756899, 4148375, 4029340, 4029805, 4195852, 4223626, 2313898, 2313900, 2313899, 4020669, 4141396, 4171675, 4337439, 4338609, 4304099, 4171077, 4329385, 4305997, 4233909, 4303797, 4330920, 44809593, 46271741¸ 44812292, 44805147, 44805244, 44805318, 44811692, 2789601-2789606, 2789613-2789616, 2785794, 2785793, 2789608-2789612, 2789595-2789600, 4065262, 40756943, 40757045, 40756830, 40756983, 40756942, 42734805, 2110624, 4197753, 2006895, 4330521, 4329659, 4330647, 2108060, 2108057, 4119901, 40756896, 4338617, 44511240, 43531439, 43531438, 43533247, 43533352, 44784573, 43531442, 43531441, 43533353, 43533248, 43531440, 37017357, 40756947, 45890619, 2313897, 4148315, 2617370, 2617369, 2313796, 4179395

CABG: 4018579, 42537532, 42539671, 42537533, 42537534, 44511088, 44511085, 44511087, 44511086, 4219321, 4148779, 4168141, 2100873, 2100872, 4146972, 42537528, 42537529, 42537530, 42537531, 4148030, 44511082, 44511079, 2725356, 2725388, 2725350, 2725382, 2725368, 2725401, 2725362, 2725395, 42894402, 42894408, 2725374, 2725407, 2725357, 2725389, 2725351, 2725383, 2725369, 2725402, 2725363, 2725396, 42894403, 42894409, 2725352¸ 2725384, 2725346, 2725378, 2725364, 2725397, 2725358, 2725391, 42894398, 42894404, 2725370, 2725403, 2725375, 2725377, 2725376, 2725390, 2725354, 2725386, 2725348, 2725380, 2725366, 2725399, 2725360, 2725393, 42894400, 42894406, 2725372, 2725405, 2725353, 2725385, 2725347, 2725379, 2725365, 272539, 2725359 , 2725392, 42894399, 42894405, 2725371, 2725404, 2725355, 2725387, 2725349, 2725381, 2725367, 2725400, 2725361, 2725394, 42894401, 42894407, 2725373, 2725406, 2724724, 2724756, 2724718, 2724750, 2724736, 2725215, 2724730, 2724763, 42894245, 42894251, 2724742, 2725221, 2724725, 27 24757, 2724719¸ 2724751, 2724737, 2725216, 2724731, 2724764, 42894246, 42894252, 2724720, 2724752, 2724714, 2724746, 2724732, 2724765, 2724726, 2724759, 42894241, 42894247, 2724738, 2725217, 2724743, 2724745, 2724744, 2724758, 2724722, 2724754, 2724716, 2724748, 2724734, 2725213, 2724728, 2724761, 42894243, 42894249, 2724740, 2725219, 2724721, 2724753, 2724715, 2724747, 2724733, 2724766, 2724727, 2724760, 42894242, 42894248, 2724739¸ 2725218, 2724723, 2724755¸ 2724717, 2724749, 2724735, 2725214, 2724729¸ 2724762, 42894244, 42894250, 2724741, 2725220, 2725294, 2725326, 2725288, 2725320, 2725306, 2725339, 2725300, 2725333, 42894233, 42894239, 2725312, 2725345, 2725295, 2725327, 2725289, 2725321, 2725307, 2725340, 2725301, 2725334, 42894234, 42894240, 2725290, 2725322, 2725284, 2725316, 2725302, 2725335, 2725296, 2725329, 42894229, 42894235, 2725308, 2725341, 2725313, 2725315, 2725314, 2725328, 2725292, 2725324, 2725286, 2725318, 2725304, 2725337, 2725298, 2725331, 42894231, 42894237, 2725310, 2725343, 2725291, 2725323, 2725285, 2725317, 2725303, 2725336, 2725297, 2725330, 42894230, 42894236, 2725309, 2725342, 2725293, 2725325, 2725287, 2725319, 2725305, 2725338, 2725299, 2725332, 42894232, 42894238, 2725311, 2725344, 2725232, 2725264, 2725226, 2725258, 2725244, 2725277, 2725238, 2725271, 42894257, 42894227, 2725250, 2725283, 2725233, 2725265, 2725227, 2725259, 2725245, 2725278, 2725239, 2725272, 42894258, 42894228, 2725228, 2725260, 2725222, 2725254, 2725240, 2725273, 2725234, 2725267, 42894253, 42894223, 2725246, 2725279, 2725251, 2725253, 2725252, 2725266, 2725230, 2725262, 2725224, 2725256, 2725242, 2725275, 2725236, 2725269, 42894255, 42894225, 2725248, 2725281, 2725229, 2725261, 2725223, 2725255, 2725241, 2725274, 2725268, 42894254, 42894224, 2725247, 2725280, 2725231, 2725263, 2725225, 2725257, 2725243, 2725276, 2725237, 2725270, 42894256, 42894226, 2725249, 2725282, 42537524, 42537525, 42537526, 42537527, 4020217, 4336464, 4337056, 4000733, 4336467, 4336465, 4339629, 4337737, 4336466, 4233421, 4305509, 4309432, 4011931, 4253805, 2107242, 2107243, 2107244, 2107231, 2107216-2107224, 2107226-2107228, 2107250, 4140107, 4018692, 44511110, 4189169, 44806690, 2108631, 4000732, 4173656, 4169964, 2721131-2721134, 44511089, 44511107, 44511083, 44511114, 44511099, 44511095, 44511077, 2617584, 43528000-43528004, 37111313, 4020213, 44511091-44511094, 4166585, 4173645, 4302815, 44511075, 44511074, 4018762, 4018693, 4233420

Cardiogenic Shock: 43752073, 43626151, 43716081, 43824151, 43608156, 43824152, 43842397, 43842398, 43608320, 43680545, 43590324, 43590325, 43608318, 43734332, 43608319, 43734333, 43844212, 43718002, 43610074, 36895563, 43808098, 43041055-43041057, 36808883, 40715090, 43718066, 43826188, 40715091, 784545-784547, 43592192, 43808100, 43682323, 43736231, 43789978, 43700347, 35146430, 43592188, 43610127, 43646322, 43592189, 43862461, 43646321, 43592191, 43826189, 43664490, 43844268, 43789975, 43789976, 43826187, 43592187, 43718067, 43664488, 43610126, 43808099, 35781970, 40715097, 35782117, 40715095, 35781607, 40715094, 35781763, 40715096, 35145595, 40715092, 35130654, 36808942, 35161636, 35149995, 40715093, 43297063, 36808949, 36808873¸36808978, 36808946, 36279454, 36279453, 36279492, 43664489, 43844267, 43700346, 43592190, 43264446, 36808924, 43259021, 36808979, 36808901¸ 36808915¸ 43736230, 43862462, 43789977, 43628206¸ 36808990, 36808895, 36808909, 36780232, 36780231¸ 42479551, 21097768, 43041060, 43041058, 43041061, 43041059, 43197165, 43186148¸ 43175288¸ 43219042, 43219043¸ 43142082, 21078184, 21166583, 21058466¸ 21097769, 42628993, 40712098-40712106, 784675-784677, 41381327¸ 41379695, 35782890¸ 35783182, 35782991, 35782935¸ 41377641, 36418515, 36418514, 41382245, 41381205, 35783321, 35783121, 35783056, 35783183¸ 21166779, 43264543, 43291768, 43259136, 21166780, 21157029, 43264542, 21078362¸ 21078363, 36280276, 36280304, 36280089, 21127303, 21088129, 21068603, 36280194, 36280309, 21058656, 21058655, 43270038, 43280940, 43280941, 21157028, 43270037, 21068602, 36779629, 36779628, 36779581, 36779627, 36779626, 36895678, 43219288, 43042088, 43042089, 43186388, 43164466, 43153457, 43219289, 43208281, 43164467, 43164468, 43208282, 43208283, 43142322, 43208284, 43219290, 41376733, 41376732, 41380496, 41378205, 41376731, 41380931, 41377482, 41380495, 41380083, 41382246, 41377643, 41380260, 41379149, 41381469, 41381468, 41377642, 41377289, 41381104, 41378773, 36895725, 43646858, 36895733, 43790481, 43736733, 43826679, 43718596, 43628758, 43790482, 43808634, 43700903, 43862990, 43718633, 43700938, 43844816-43844818, 43610681, 43790523, 43736763, 43665015, 1719037, 42628995, 35157776, 35156308, 35131472, 35138132, 35137511, 35139385, 42918864, 42918865, 19076658, 19076659, 19076660, 1719039, 35781054, 35780190, 42921143, 42921145-42921148, 35780906, 35781073, 35781161, 35780135, 21087818, 21028967, 21097632, 21176207, 21176208, 21146721, 793740, 41393534, 41394939, 41399260, 41391798, 41396721, 35155391, 35151437, 40713261, 40713260, 40713259, 36895987, 43665953, 41394787, 43593646, 41398226, 43665954, 43683798, 43719506, 43827632, 43719505, 43809594¸43863942, 43827631, 43611622, 43791416, 35136494, 35151009, 41390816, 35783954, 35784393, 41390815, 41396722, 41399631, 41400161, 41397854, 41391135, 36505689, 35783916, 21088391, 21108077, 21167030¸21098238, 36895964, 41392179, 43701850, 41393367, 43611623, 43593644, 43647840, 43863941, 43629742, 43683800, 43755622, 43863944, 43809595, 43737657, 43683796, 43737656, 43809593, 43827630, 43593645, 43629740, 43827629, 43611621, 43629741, 43683797, 41399261, 41397174, 43863943, 43611624, 43647841, 43683799, 35158314, 35137376¸ 35784320, 35784082, 21088386, 42628996, 42918399¸42918400, 42918397, 42918398, 43867625, 43741388¸43723256, 4163717, 46257441¸37206602, 2787811, 4315404, 4163287, 21499478, 198571, 4306699, 42940589, 42940590, 40920618¸ 40836610, 41242399, 41314918, 40940557, 41078944, 43658003¸ 43621728, 43711752, 43819774, 42940586-42940588, 40729349-40729353, 35134428, 35135654, 35132382, 35130722, 35137836, 40729357-40729360, 35131771, 35133628, 35135014, 35131994, 35148466, 35138293, 35149148, 35137169, 36888224¸ 36888783, 43585709, 43693863, 41117505, 43765634¸ 43801644, ,43855880, 43783797, 43693864, 43819776, 43765633, 783671, 783672, 41086264, 41054692, 35770798, 36420032, 41065123, 36420031, 41065122, 21034043, 43296116, 21102870, 36270739, 36263103, 44034760, 44037743, 42480389, 21092959, 36263102, 44089473, 44037745, 44037744, 36784347-36784350, 36888784, 43140098, 43140099, 43216973, 43195141, 43216974, 43195142, 43151053, 43162223, 43184114, 43151051, 43151052, 43206061, 43173260, 36277020, 44067799, 36879551, 41117506, 41023456, 41086265, 40836611, 41148974, 41148975, 41023457, 41304347, 41242402, 41127954, 40971510, 40909243, 41314920, 40971509, 41127953, 41096693, 40940560, 41065126, 41065125, 36894661, 43585708, 36889782, 43747670, 43621729, 43658004, 43621730, 43639877, 43639876, 41078945, 40829284, 42940583, 42940584, 42940585, 43274564, 40929935, 40961237, 40886859, 41054691, 41023455, 41023454, 41096692, 41190567, 41042529, 41252710, 40940559, 41127951, 40971507, 41159465, 40929934, 40898812, 41323601, 44181216, 44181215, 44182724, 43290740, 43279898, 41047541, 43285341, 36690845, 36879550, 36883927, 43765632, 43765631, 35157883, 35129469, 35146517, 35161005, 35132820¸ 35138418, 35138533, 36880521, 41242401, 35749850, 41304346, 41180064, 40971508, 41127952, 42483347, 21102869, 41172798, 36892183, 36882933, 41242400, 43801645, 43621731, 43765630, 43819775, 43747671, 43621732, 43693865, 43837869, 43693861, 43801643, 43693862, 43855879, 43729730, 41159462, 40847099, 43711753, 43676121, 43819777, 43603788, 41172797, 43603789, 35749849, 42940591-42940594, 35758640, 35754525, 42940595, 42482745, 21112688, 21122318, 21053745, 42940596-42940603, 21171586, 35147214, 35160461, 35160270, 35154931, 42940605, 35138268, 43747668, 41082183, 43603787, 43765629, 43819773, 783670, 35770531, 42940606, 41236530, 44179700, 41207525, 36420033, 43747669, 36261962, 44044328, 42480387, 21073484, 36784352, 36784351, 36879865, 43206060, 43184112, 43184113, 43173259, 35150908, 37499148, 1531631, 46257511, 46257682, 38001119-380011, 3186728, 2001567, 2100812, 4336750, 4191601¸ 4338594, 4336749, 2001557, 4337306, 2001562¸ 2107498, 2107496, 2107494, 4060257, 4331380, 40485320, 2107514, 43527920, 43527921, 2107501, 2107500, 40756824, 4044874, 4142475, 4222272, 4226988, 4172818, 36885798, 35749893, 43622828, 43784879, 43802796, 783083, 783084, 43838955, 43748770, 43748769, 35132643, 43802797, 43622830, 43586851, 43820918, 43820919, 43677185, 43622829, 43857023, 43604946, 35758740, 40742759, 35746250, 40742758, 21160475, 43280028, 36814674, 44073091, 44101624, 36262752, 36265329, 43766782¸43694996, 43280029, 36811509, 43285485, 36813521, 44088606, 43857024¸43694997, 44036880, 44075762, 44075761, 36787298, 36787297, 42481305, 21170276, 43150093, 43139156, 43150094¸ 43183196, 21140687, 21111300¸ 44092945¸ 43183195, 43712831, 43216059, 35144125, 43604945, 43802795, 43659176, 43641047, 40742760, 21042606, 43712832, 4139214, 4161976, 2107499, 2107497, 2107495, 4243758, 4241906, 43527922, 40757060, 40756954, 40756900, 43527923¸45763688, 4020229, 4337304, 37206601, 1337720, 1337749¸ 19076657, 1337750, 1719042, 1337748, 37499144, 37499149, 1719038, 37499146, 1337753, 1719040, 1337751, 1719041, 19091005, 19065473, 36224130, 40035704, 37499147, 1719036, 42628992, 42628994

Respiratory failure: 1531630, 2007912, 2106469, 2108641, 2108642, 4013354, 4026054, 4056812, 4058031¸ 4072503, 4072504, 4082243, 4119642, 4140765, 4179373, 4283807, 4287921, 4331311, 4335481, 4335584, 4335585, 4337045, 4337046, 4337047, 4337048, 4337615, 4337616, 4337617, 4337618, 4339623, 37116698, 40487536, 44515633, 46257510, 46257543

History of CABG: Includes all the codes as “CABG” as well as 443563, 761735, 4163883, 4252385, 35615052, 35615053¸37108804, 37110242, 37118984, 40481132, 40481133, 40482638, 40482655, 40483189, 43021857, 43021858

History of Stroke: 36684840, 4031045, 4045747, 763094, 40484101, 36685012, 36685003, 42535344, 42535687, 764847 , 42535411, 42535703, 36685004, 761798, 759831, 4111710, 3184799, 37396293¸ 4045734, 3188623, 3187113, 4211509, 36716999, 4111711, 3184775, 43531607, 3188677, 37312016, 37312017, 37110239, 37312015, 37312014, 37110238, 37110678, 37110237, 37110679, 37312013, 381316, 36309723, 37395576, 37309665, 761785, 42539262, 42535148, 42535508, 42535112, 42535504, 42535146, 42539195, 42535506, 761835, 42535461, 765281, 42535149, 42535507, 42535111, 42535503, 42535147, 42535110, 42535505, 761836, 37395574, 37395575, 42535460, 37209562, 37309657, 42535459, 42535458, 42535511, 42539166, 42535114, 42535512, 761789, 42535113, 761792, 762344, 764721, 765568, 4337830, 42535706, 42535681, 4099974, 44790196, 42535412, 36685011, 36685002, 443465, 42535232, 42535707, 42535679, 4153352, 4090122, 42535415, 764817, 4078314, 4145413, 35609033, 40481389, 40484910, 42535677, 764853, 764816, 42535704, 42535676, 764815, 4179392, 46270602, 4077982, 37209667, 764952, 44783354, 4208306, 4331077, 37209666, 43530672, 43530627, 765814, 42535013, 36712799, 43530667, 43530668, 762807, 765815, 37016190, 37108825, 43531606, 43531603, 43531615, 4131383, 4310996, 43530669, 43530670, 37110765, 4219010, 36716860, 762340, 761797, 761791, 3176312, 761793, 4112022, 761795, 762345, 37309662, 37309661, 4077819, 42535686, 42535419, 197303, 765518, 443525, 764851, 764814, 762089, 762093, 762090, 42535417, 42535416, 764849, 764820, 40480946, 764813, 40482266, 764850, 765461, 765462, 42539180, 762091, 764852, 379778, 37395562, 444091, 443790, 443864, 377254, 4045742, 4159152, 4198778, 4006295, 761790, 43531605, 4189462, 761799, 765283, 3185422, 4023571, 372654, 4146293, 43530612, 43530611, 443609, 443599, 43530742, 42534989, 36716757, 4298750, 4045748, 763095, 4301259, 3188894, 4238315, 40482301, 762339, 3174092, 761796, 3174271, 3174415, 4110196, 761794, 762351, 764707, 43530665, 43530736, 43530623, 43531592, 36717605, 43531610, 40481354, 42535705, 42535689, 42535690, 36210384, 37110241, 4168056, 4046363, 4097325, 4300312, 40480273, 4159140, 4078315, 440426, 42535684, 764848, 36684470, 44782753, 43531595, 43530744, 42535702, 42535688, 42534983, 42539260, 4086178

Controlled Type 2 Diabetes: 4321756, 36717156, 43531588, 45769888, 4196141, 37016768, 45763582, 40483315, 4221495, 43531578, 43531559, 43531566, 43531653, 43531577¸43531562, 37309630, 45769894¸43531616¸36684827, 45757474, 37018912, 443732, 43531597, 443733, 376065¸ 43531564, 45757280, 45769906, 4177050, 4223463¸ 43530690, 4222876, 37018728, 45772019, 37016349, 45770880, 201530, 4215719, 45771064, 45757444-45757447, 45757363, 45772060¸ 36714116, 45769875, 4130162, 45757075, 761062, 45771072, 443734, 4228443, 4140466, 45770830, 35626070, 45769905, 45757435, 43531651, 45770881, 4222415, 4099216¸45769828, 43531563¸45757450, 37312203, 37312202, 45770883, 37016354, 43530656¸45769836¸443729, 43530689, 45757278, 4221487, 4223739, 37017432¸ 3192767¸ 3191208, 3194332, 4063043, 43531010, 4129519, 43530685, 45770831, 45757499, 443731, 45770928, 4226121, 45769872, 45769835, 761053, 36712670, 46274058, 4142579, 45770832, 45773064, 45757508, 4230254, 4304377, 40485020, 4198296, 4200875, 4099651, 4193704, 45766052, 45769890, 37312205, 36712686, 45757277, 37312204, 36712687, 45757449, 43531608

Poorly Controlled Type 2 Diabetes: 3193274, 3192767, 3194082, 3191208, 3194332, 40482801

Controlled Type 1 Diabetes: 36715571, 45769891, 37016767, 45763585, 4128019, 4225656, 45773688, 45773576, 45769901, 45771075, 45769902, 45769903, 45769837, 35626765, 45769832, 45757674, 435216, 42538169, 42535539, 377821, 37016353, 45769904, 43531565, 4221344, 4223303, 37017429, 37016348, 45757432, 443592, 201531, 42535540, 45771067, 45769876, 4228112, 45757362, 4047906, 4102018, 36717215, 45757073¸ 439770, 4224254, 4145827, 4143857, 35626069, 45757535, 37016179, 43530660, 37016180, 4225055, 4224709, 45769829, 45769830, 37312218, 45763583, 45769834, 36713094, 318712, 37018566, 4222687, 4222553, 37017431, 3198350, 3196797, 3192955, 3192052, 4063042, 43531008, 43531009, 45763584, 45757604, 200687, 45757266, 4227210, 45771533, 45773567, 45769833, 765373, 46269764, 4143689, 45769873, 4099215, 40484649, 4152858, 4099214, 45766051, 45757507, 45769892, 37312201, 45770902, 37312200, 45757074

Poorly Controlled Type 1 Diabetes: 3194119, 3198350, 3196797, 3192955, 3192052, 40484648

Hyperlipidemia: 4144326, 4294296, 45757265, 35608140, 4159131, 37016353, 43531564, 45757280, 4030618, 4030586, 4143177, 4079876¸ 4029890, 45757500¸ 4134862, 4029259, 4079885, 4295609, 4295608, 4298010, 437521, 4142496, 4029262, 4301409, 4300461, 4104485¸ 4144529, 4220010, 4029261, 36715325, 4079886, 4029305, 36674388¸ 432867, 37016144, 45757432, 45770880¸ 4298723, 4096215, 4120314, 4030619, 438720, 43530660, 43531651, 4029258, 40482885, 4270878, 4223495, 4291436, 4029263, 4298733, 4294297¸ 4031945, 4029260, 437827, 440360, 4079887, 4029891, 4292672, 4029892, 4299409, 36676683, 4031947

Controlled Hypertension: 312648, 4215640, 4034031, 4148205, 4269358, 4028741, 314958, 4249016, 4167358, 42538697, 2101836, 320128, 4083723, 4302591, 4321603, 4217486, 4179379, 4048212, 4017170, 4058987¸ 4322735, 45771067, 45771064, 45757444-45757447, 4061667, 2108558, 4108213, 4110948, 4178312, 4094374, 4174979, 4219323, 4006325, 38001132, 38001133, 4262182, 44783643, 4276511, 4159755, 44783644, 36713024, 4263067, 317898, 4110947, 4201850, 4212496, 762994, 45757787, 4305599, 443771, 4032952, 317895, 4253928, 44809026, 44811933, 44811932, 44809569, 4242878, 4180283, 4209293, 4199306

Uncontrolled Hypertension: 44809548-45768449, 43020424, 40481896, 317898, 4289933, 4023318, 4162306, 4028951¸4218088, 318437, 4110947, 37208293¸ 4016922, 37208172, 44809027

Heart Failure with Reduced Ejection Fraction: 44782718, 44782733, 40480602, 40480603, 44782719, 40479192, 40482727, 4139864, 45766164-45766167, 45773075, 764873, 764871, 764872, 443580, 43530642, 36717359, 43020421, 36712929, 43021840

Heart Failure with Preserved Ejection Fraction:44782718, 40481042, 44782733, 40481043, 44782719, 40479576, 40482727, 443587, 43530643, 43021842, 43021841

Peripheral Arterial Disease: 4348319, 42536636, 37109921, 37109923, 42536634, 37116421, 44782819, 4195973, 4195971, 4194886, 4193056¸ 4117933, 4199183, 37110251, 44808832, 36717256, 4329498, 44808745, 35615076, 35615075, 44808747, 4137551¸ 4137550, 36713012, 35615107, 434961, 44808833, 44813823, 4143588, 36713013, 35615106, 4141975, 44808746, 36308312, 764026, 4193057, 4030663, 4195972

Obesity: 4271317, 4171972, 4270189, 4079899, 4060985, 4256640, 4235799, 4087487, 40481140, 36713437, 36678790, 4097929, 4097996, 4182506, 4100857, 4160821, 4029277, 4029276, 4029900, 36717154, 4005991, 4185912, 4163032, 4171147, 380500, 37310479, 37310480, 4177337, 4220527, 4203289, 35622038, 36674490, 36674893, 4171317, 438731, 37208175, 36716144, 37110069, 37395980, 42872398, 439893, 4216214, 434005, 4037679, 4215968, 763588¸ 44811711, 44811712¸ 37311728, 44811757, 433736, 4212443, 4189664, 4215969, 4189665, 36716555, 37204685, 37204815, 37206117, 37397209, 36716151, 37311904, 45757112, 4183240, 36717199, 4093860, 36674827, 3199162, 45771307, 36676689, 37204691, 37018860, 42539192, 4217557, 4211019, 36714072, 36714548

Atrial Fibrillation or Flutter: 45883018, 313217, 4108832, 44782442, 45883019, 314665, 36712986, 4141360, 4137382, 4117112, 4064452, 4065288, 37395821, 4323077, 37395937¸ 4119601, 45768480, 4119602, 4154290, 4146580, 4232691, 4232697, 42539346, 4199501, 36714994

History of CAD: 4119951, 43531588, 36712983, 4199962, 764123, 4242670, 4178321, 317576, 46269996, 4175846, 37016181, 37309630, 40481919, 3180023, 44806109, 4108673, 43020660, 4155962, 764149, 4155007, 36714444, 4168972, 4178622, 4161455, 4111393, 4124682, 36712982

Current Smoker: 45757366, 4036561, 4043057, 4036555, 4036556, 4186872, 4044778, 4038735, 4038737, 4038736, 4036091, 4038738, 4218741, 4043060, 4047454, 4246415, 4041508, 4269183, 764103, 764104, 4276526, 4052466, 42536336, 4239437, 4238769, 37017812, 4036560, 4038734, 4036085, 4041511, 4052947, 4209006, 762499¸ 4052029, 4042037, 762498, 4131520, 4052030, 4044776, 4209585, 4043054, 4193014¸ 35610375, 36684331, 44811339, 4044775¸ 37395605, 4218917, 4041509, 44804436, 3193708, 45757454, 4293731, 4058138, 4298794, 4206526, 4308496, 4269046¸ 4216476, 4204653, 4141787, 44804570, 4308498, 4308499, 4305744, 4038731, 4036084, 4043053, 4043061, 4043050, 4036086, 4036558, 45757459, 4144271, 4041306, 4005823, 4043062, 4036089, 4036083, 4036087, 4144273, 4043056, 37017610, 4044777, 4058136, 44809281

Former Smoker: 4232375, 44802113, 4052949, 4092281, 4148416, 4141783, 35610343, 4145798, 35610347, 4141782, 35610345, 4052465, 4310250, 35610339, 42536346, 46270534, 4043059, 45765917, 4148415, 35610349, 4141784, 35610340, 762500, 762501, 4237385, 4207221

Body Weight: 3025315, 4099154, 46234683, 40759198, 21492642, 3026600, 3013762, 3013853, 3009617, 3022281, 3005422, 40761330, 3010220, 3011054, 3023166, 3005778, 4178502, 4310154, 4175354, 42536495, 0-7, 40484200, 3026659, 3027492, 3013747, 18584, 37205098, 37206608, 18833, 9

Body Height: 3036277, 3014149, 3015514, 3023540, 3019171, 4177340, 2, 7, 5, 3¸ 9

COVID-19 Test: 0-9, 19, 37310257, 757685, 723477, 706166, 586523, 586518, 706174, 706167, 706157, 706155, 715272, 757678, 706161, 586524¸ 586525, 586520, 706175, 706156, 706154, 723469, 706168, 723478, 723464, 586528, 586529, 715262, 723476, 586526, 757677, 706163, 715260, 715261, 723463, 706170, 706158, 706169, 723471, 723470, 706160, 706173, 723467, 723468, 723465, 586519, 723466, 586516, 706172, 706171¸706165¸723472, 706159

COVID Positive Test: 45877985, 4126681, 11882, 0, 5, 9191, 45884084, 36715206, 45881802¸15255, 8

Cancer: 138708, 135496, 134305, 141816, 4079686, 4189938, 135762, 40483761, 40481524, 140352, 4003184, 4002497, 4137687, 4003188, 37396742, 4299149, 40488896, 4003021, 4079282, 4173963, 4082311, 434592, 4173974, 4041800, 4097560, 193428, 4091768, 4097561, 196055, 315481, 439293, 435758, 437504, 40481907, 437238, 443565, 432271, 193155, 198100, 140057, 134597, 133438, 134603¸132572, 4133599¸4082487, 6¸ 4300704, 4003830, 4001328, 4002356, 1, 138099, 4139358, 4094548¸ 4212994, 40482893, 4147411, 45765770, 4003833¸4001329, 4002357, 4170421, 40486465¸ 40490328, 40492267, 40492266, 4038845, 4217892, 4288751, 4001172, 4002494, 4038839, 4038835, 436651, 200355, 195761, 440965, 434877, 437818, 436652, 316356, 198704, 4041798, 432267¸439285, 439282, 141243, 442151, 433161, 442150, 313980, 439281, 438373¸ 201242, 194876, 440351, 438703, 442163, 442162, 321522, 198381, 4038843, 435203, 192268, 194594, 136655, 434299, 442161, 442160¸ 315202, 442159, 4038842, 434302, 193434, 199760, 136928, 314600, 442158, 442157, 315763, 193157, 4003836, 4001664, 40482847¸ 434584, 198085, 254583, 312846, 140958, 258981, 375479, 4038846, 4044708, 40481357, 317510, 135766, 4001171¸ 4038841¸132853, 134596, 40482859, 37017321, 37018934, 46271402, 46271647, 4003834, 440058¸ 200349, 195195, 435753, 441521, 438698¸ 192560, 132841, 80665, 141524, 133710, 4247719, 433975, 195482, 200051, 435493, 81237, 200343, 4094872, 138377, 444224, 441513, 442134, 25486, 134290, 436635, 261236, 4040379, 4095168, 194593, 4003684, 36684817, 4003028, 192847, 199754, 139750, 436353, 4095589, 4092513, 135764, 196645, 36684820, 441800, 436913, 433423, 4157456, 252840, 4089860, 4092515, 442168, 432558, 256633, 42709931, 198985, 442122, 136917, 432260, 4151263, 37018875, 40492268, 438691, 194589, 434587, 437501, 196047, 136915, 201517, 4095592, 4094863, 140666, 4001170, 196360, 435752, 441802, 139753, 4312685, 200054, 141232, 4110889, 132850, 192261, 434293, 441805, 372567, 200963, 133147, 76924, 4244051, 4111023, 140967, 4003027, 79749, 133420, 436352, 438086, 440658, 196049, 434590, 435754, 132575, 4002343, 193422, 4247238, 26052, 434588, 192836, 436923, 133713, 443391, 4054513, 438090, 4162860, 80340, 440344, 4246141, 254282, 76349, 4244488, 443382, 4180312, 198092, 76914, 26638, 438692, 198988, 376647, 200052, 4153890, 4114222, 4160342, 199747, 4246802, 140046, 440345, 195483, 193719, 196359, 4149851, 4147164, 4130672, 378081, 4156114, 436358, 434292, 4003179, 136639, 45770892, 4032870, 4095312, 4131761, 197803, 380055, 40486896, 434289, 4003175, 135491, 434285, 4111917, 4114198, 4308811, 436043, 441806, 374874, 4155171, 28083, 134295, 196048, 197506, 4089665, 434300, 4002498, 4003674, 201801, 201519, 26361, 193418, 438360, 77812, 443381, 0, 4003029, 4162253, 137809, 436640, 24296, 437798, 437220, 4091467, 443380, 194878, 434881, 4187851, 201238, 440335, 78093, 196044, 133969, 4091490, 443384, 200338, 133154, 4188545, 135750, 432263, 436922, 259755, 132565, 4095018, 4114221, 198088, 760936, 4158563, 441233, 201518, 435474, 439738, 138074, 4089777, 40481901, 435492, 133158, 4187850, 132258, 4157454, 435487, 441223, 75488, 201231, 40479608, 320347, 4216139, 432264, 435751, 441515, 436344, 261514, 261808, 4092358, 4002496, 198374, 4301668, 134579, 197806, 193138, 439739, 432843, 195197, 4092235, 4264693, 4038838, 36716501, 432838, 4179720, 257503, 40650479, 433976, 40481522, 4095748, 4225982, 40487528, 439392, 27235, 438370, 4247836, 440339, 433716, 11, 73153, 22, 133419, 432254, 4246029, 260336, 135489, 200962, 197799, 3, 436045, 23, 198695, 259748, 432837, 437224, 197507, 438089, 438368, 443719, 4246127, 4033836, 198082, 4162115, 4247822, 140950, 379756, 192255, 133424, 40488812, 4095432, 4100425, 4033318, 198104, 432845, 438694, 137800, 435190, 437498, 4115271, 4334322, 4033891, 4003693, 4162859, 433149, 442131, 438693, 438699, 31509, 4041799, 4091464, 4001666, 258375, 441225, 436926, 440649, 4002340, 74582, 433709, 135759, 4091469, 45767695, 79740, 436348, 432848, 316644, 4247336, 195480, 440047, 439267, 4092223, 321526, 25748, 436643, 378696, 438979, 256646, 4311499, 432262, 439270, 4091486, 437233, 438095, 432844, 4246808, 434577, 437805, 377811, 432257, 439265, 4091621, 436059, 133711, 438982, 197225, 439746, 4247842, 433704, 435484, 138378, 4097284, 4040380, 196051, 436042, 4003675, 4311480, 438367, 198091, 4312698, 135765, 4094262, 135204, 140955, 438080, 197500, 435485, 432559, 438977, 436054, 439266, 4094260, 201813, 321234, 80045, 255192, 132832, 4246137, 441520, 435478, 140664, 4095892, 442169, 440044, 433143, 373151, 137219, 439404, 79758, 440956, 195760, 4097283, 134879, 22839, 444203, 375490, 138351, 432833, 434880, 81239, 4079683, 9, 4098597

**eTable 1: Baseline Demographics Pre and Post Matching**

|  | **All treatment arms** | **Coronary angiogram with PCI, without CABG: number (%)** | | **Coronary angiogram with PCI, with CABG: number (%)** | | **Coronary angiogram without PCI, without CABG: number (%)** | | **Coronary angiogram without PCI, with CABG: number (%)** | |
| --- | --- | --- | --- | --- | --- | --- | --- | --- | --- |
| **Matching** |  | **Pre** | **Post** | **Pre** | **Post** | **Pre** | **Post** | **Pre** | **Post** |
| **Total patients** | 10506 | 3743 | 579 | 1487 | 203 | 3840 | 763 | 1436 | 194 |
| **Diabetes** | 4470  (42.5) | 1640 (43.8) | 272 (47) | 578 (38.9) | 94 (46.3) | 1601 (41.7) | 296 (38.8) | 651 (45.3) | 91 (46.9) |
| **HLD** | 7523 (71.6) | 2844 (76.0) | 448 (77.4) | 1095 (73.6) | 142 (70.0) | 2514 (65.5) | 437 (57.3) | 1070 (74.5) | 163 (84.0) |
| **HTN, controlled** | 960 (9.1) | 347 (9.3) | 49 (8.5) | 91 (6.1) |  | 418 (10.9) | 52 (6.8) | 104 (7.2) |  |
| **HTN, poorly controlled** | 7451 (70.9) | 2766 (73.9) | 430 (74.3) | 986 (66.3) | 140 (69.0) | 2706 (70.5) | 494 (64.7) | 993 (69.2) | 145 (74.7) |
| **HFrEF** | 2678 (25.5) | 752 (20.1) | 116 (20.0) | 356 (23.9) | 48 (23.6) | 1248 (32.5) | 225 (29.5) | 322 (22.4) | 54 (27.8) |
| **HFpEF** | 1922 (18.3) | 596 (15.9) | 92 (15.9) | 193 (13.0) |  | 906 (23.6) | 144 (18.9) | 227 (15.8) | 41 (21.1) |
| **Obesity** | 4675 (44.5) | 1647 (44.0) | 298 (51.5) | 645 (43.4) | 84 (41.4) | 1728 (45.0) | 405 (53.1) | 649 (45.2) | 104 (53.6) |
| **Atrial fibrillation** | 2173 (20.7) | 632 (16.9) | 106 (18.3) | 264 (17.8) | 34 (16.7) | 936 (24.4) | 178 (23.3) | 341 (23.7) | 72 (37.1) |
| **COPD** | 2858 (27.2) | 1014 (27.1) | 196 (33.9) | 329 (22.1) | 56 (27.6) | 1229 (32.0) | 239 (31.3) | 286 (19.9) | 45 (23.2) |
| **CAD** | 8002 (76.2) | 3034 (81.1) | 477 (82.4) | 1294 (87.0) | 179 (88.2) | 2472 (64.4) | 431 (56.5) | 1202 (83.7) |  |
| **Prior CABG** | 2138 (20.4) | 289 (7.7) | 32 (5.5) | 1128 (75.9) | 153 (75.4) | 265 (6.9) | 60 (7.9) | 447 (31.1) | 59 (30.4) |
| **Prior stroke** | 421 (4.0) | 145 (3.9) |  | 49 (3.3) |  | 165 (4.3) | 31 (4.1) | 44 (3.1) |  |
| **Cancer** | 1149 (10.9) | 409 (10.9) | 66 (11.4) | 124 (8.3) |  | 487 (12.7) | 71 (9.3) | 129 (9.0) |  |
| **Current smoker** | 2820 (26.8) | 976 (26.1) | 136 (23.5) | 406 (27.3) | 55 (27.1) | 908 (23.6) | 139 (18.2) | 353 (24.6) | 41 (21.1) |
|  | **All treatment arms** | **Coronary angiogram with PCI, without CABG: number (%)** | | **Coronary angiogram with PCI, with CABG: number (%)** | | **Coronary angiogram without PCI, without CABG: number (%)** | | **Coronary angiogram without PCI, with CABG: number (%)** | |
| **Former smoker** | 555 (5.3) | 185 (4.9) | 27 (4.7) | 113 (7.6) |  | 156 (4.1) | 32 (4.2) | 79 (5.5) |  |
| **Male** | 6694 (63.7) | 2525 (67.5) | 398 (68.7) | 1092 (73.4) | 162 (79.8) | 2035 (53.0) | 441 (57.8) | 1042 (72.6) | 166 (85.6) |
| **Female** | 3808 (36.2) | 1216 (32.5) | 181 (31.3) | 394 (26.5) | 41 (20.2) | 1805 (47.0) | 322 (42.2) | 393 (27.4) | 28 (14.4) |
| **Asian** | 182 (1.7) | 63 (1.7) |  | 35 (2.4) | 0 | 50 (1.3) |  | 34 (2.4) |  |
| **Black or African American, non-Hispanic** | 1491 (14.2) | 470 (12.6) | 64 (11.1) | 142 (9.5) | 26 (12.8) | 733 (19.1) | 154 (20.2) | 146 (10.2) |  |
| **Hispanic or Latino** | 619 (5.9) | 210 (5.6) | 57 (9.8) | 97 (6.5) |  | 220 (5.7) | 71 (9.3) | 92 (6.4) |  |
| **White, non-Hispanic** | 7495 (71.3) | 2748 (73.4) | 437 (75.5) | 1109 (74.6) | 152 (74.9) | 2568 (66.9) | 508 (66.6) | 1070 (74.5) | 161 (83.0) |
| **Other, non-Hispanic** | 33 (0.3) |  | 0 |  | 0 | 21 (0.5) |  |  |  |
| **Unknown** | 686 (6.5) |  |  |  |  | 248 (6.5) | 24 (3.1) |  |  |
